# High salt intake and HIV infection on endothelial glycocalyx shedding in salt-sensitive hypertension

**DOI:** 10.3389/fcell.2024.1395885

**Published:** 2024-07-16

**Authors:** Sepiso K. Masenga, Situmbeko Liweleya, Annet Kirabo

**Affiliations:** ^1^ HAND Research Group, School of Medicine and Health Sciences, Mulungushi University, Livingstone, Zambia; ^2^ Department of Medicine, Vanderbilt University Medical Center, Nashville, TN, United States; ^3^ Vanderbilt Center for Immunobiology, Nashville, TN, United States; ^4^ Vanderbilt Institute for Infection, Immunology and Inflammation, Nashville, TN, United States; ^5^ Vanderbilt Institute for Global Health, Nashville, TN, United States

**Keywords:** salt, sodium, hypertension, salt-sensitive hypertension, HIV, glycocalyx, endothelial glycocalyx

## Abstract

The endothelial glycocalyx is closely associated with various physiological and pathophysiological events. Significant modification of the endothelial glycocalyx is an early process in the pathogenesis of cardiovascular disease. High dietary salt and HIV infection damages the endothelial glycocalyx causing endothelial dysfunction and increasing the risk for salt-sensitive hypertension and cardiovascular disease. The two factors, HIV infection and dietary salt are critical independent predictors of hypertension and cardiovascular disease and often synergize to exacerbate and accelerate disease pathogenesis. Salt-sensitive hypertension is more common among people living with HIV and is associated with risk for cardiovascular disease, stroke, heart attack and even death. However, the underlying mechanisms linking endothelial glycocalyx damage to dietary salt and HIV infection are lacking. Yet, both HIV infection/treatment and dietary salt are closely linked to endothelial glycocalyx damage and development of salt-sensitive hypertension. Moreover, the majority of individuals globally, consume more salt than is recommended and the burden of HIV especially in sub-Sahara Africa is disproportionately high. In this review, we have discussed the missing link between high salt and endothelial glycocalyx shedding in the pathogenesis of salt-sensitive hypertension. We have further elaborated the role played by HIV infection and treatment in modifying endothelial glycocalyx integrity to contribute to the development of hypertension and cardiovascular disease.

## 1 Introduction

The endothelial glycocalyx is a carbohydrate-protein-rich layer that lines the surface of the whole vascular endothelium (Reitsma et al., 2007). The endothelial glycocalyx plays a crucial role in vascular homeostasis and has been implicated in the initial development of cardiovascular disease processes (Kim et al., 2017). The endothelial glycocalyx is the main buffer for sodium ions in the vasculature (Oberleithner, 2015; Oberleithner and Wilhelmi, 2015). Degradation or shedding of the endothelial glycocalyx is one of the first steps that is linked to endothelial dysfunction (Li and Bonventre, 2016). A damaged glycocalyx is a pathological feature underlying salt-sensitive hypertension and many forms of cardiovascular disease (Li and Bonventre, 2016).

Chronic high dietary salt is an independent predictor of salt sensitive hypertension, stroke, heart attack and death (Nista et al., 2020). The underlying mechanism behind salt-induced hypertension and cardiovascular disease is related in part to sodium handling in the vascular bed. High salt has been implicated to induce endothelial glycocalyx shedding leading to Na^+^ and Ca^2+^ overload, Na^+^ extravasation into the interstitial space, epithelial cell damage, vascular smooth muscle cell hypertrophy and vascular inflammation (Oberleithner et al., 2007; Oberleithner and Wilhelmi, 2015; Schierke et al., 2017; Bkaily et al., 2018; Weinbaum et al., 2021; Sembajwe et al., 2023). In individuals with salt-sensitive hypertension, endothelial glycocalyx shedding may be more pronounced (Choi et al., 2015; Vinaiphat et al., 2023).

Salt-sensitive hypertension is defined as a significant increase in blood pressure that mirrors an increase in salt intake in persons with hypertension (Elijovich et al., 2016). Salt-sensitive hypertension is a major risk for cardiovascular and cerebrovascular adverse events (Elijovich et al., 2016; Bailey and Dhaun, 2024) and is more common in Black people and among persons living with HIV (PLWH) as reported from a study in Zambia (Elijovich et al., 2016; Masenga et al., 2021). HIV-infection and the use of antiretroviral therapy (ART) increases the risk for hypertension development and cardiovascular disease (Masenga et al., 2019). There is some emerging evidence of a link between HIV infection and damaged endothelial glycocalyx that predisposes PLWH to many cardiovascular diseases and hypertension. However, the use of ART likely ameliorates and restores endothelial glycocalyx integrity (Fragkou et al., 2023). There is little known about the role that HIV and ART plays in the salt-endothelial glycocalyx shedding axis in salt-sensitive hypertension. Unfortunately, there are no studies known to us that provide the link between salt intake, HIV infection and shedding of the endothelial glycocalyx so we will discuss the contribution of salt separate from that of HIV infection with a notion that the two factors synergize to exacerbate hypertension. However, we recently demonstrated that PLWH who are salt sensitive and have hypertension consumed more salt than the HIV negative and had a pressor response to salt that was more pronounced than the HIV negative group (Masenga et al., 2021). We however did not determine endothelial glycocalyx integrity in this cohort, limiting our understanding on the interplay between dietary salt and treated HIV infection on endothelial glycocalyx integrity.

Although the glycocalyx has been implicated in hypertension and cardiovascular disease, the pathophysiological and mechanotransduction mechanisms linking high salt to damaged endothelial glycocalyx in hypertension, let alone PLWH, are not clear. In this review, we have summarized the link between high salt and endothelial glycocalyx shedding and have elaborated in detail, the pathogenesis of salt-sensitive hypertension based on a damaged glycocalyx, and the role played by HIV/ART. We have further given insights on the current therapeutic modalities aimed at restoring the endothelial glycocalyx to prevent the risk for cardiovascular disease development.

## 2 The composition, structure, and role of endothelial glycocalyx

**FIGURE 1:**
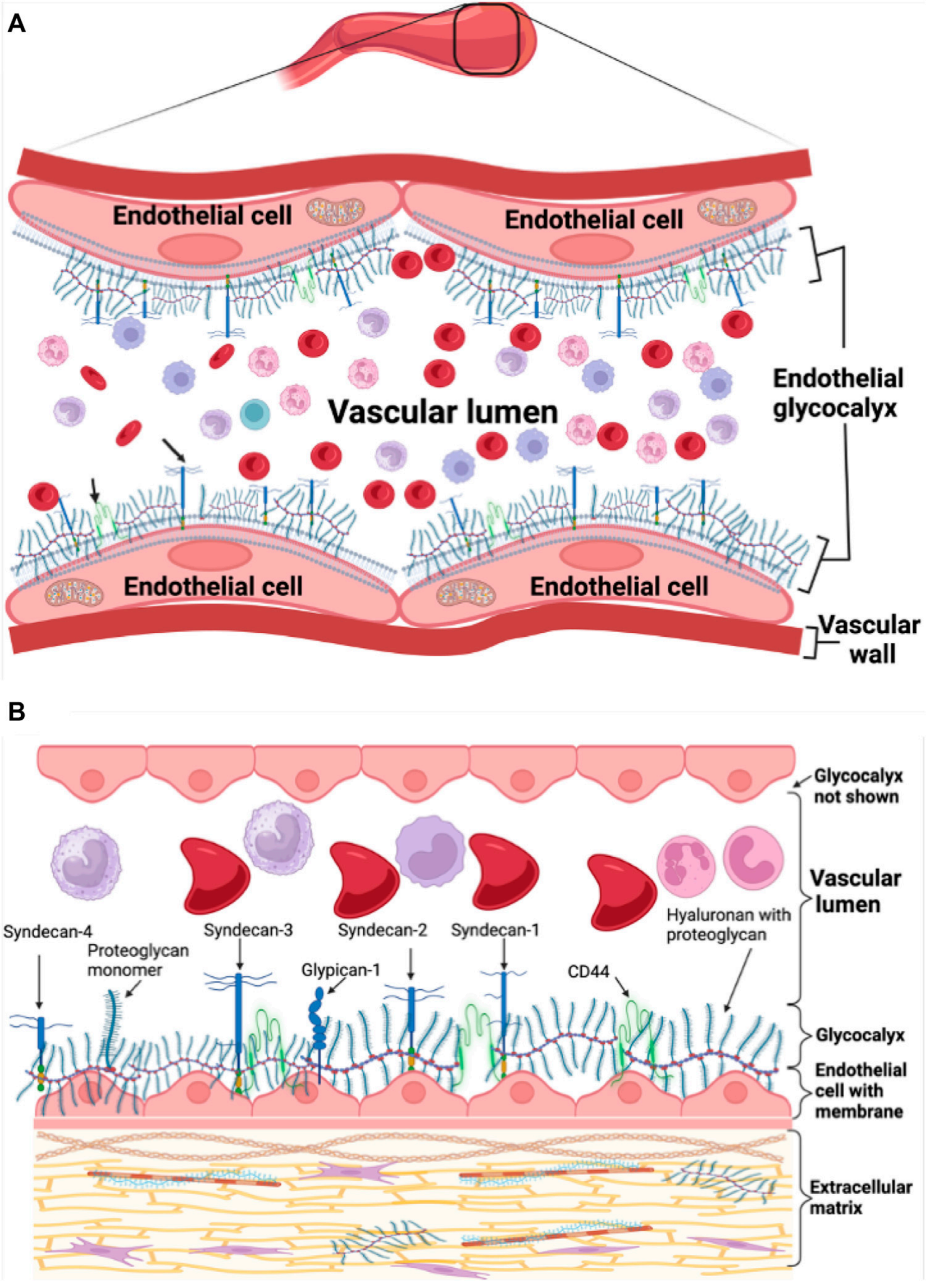
Endothelial glycocalyx schematic representation. **(A)** Healthy endothelial glycocalyx linked to endothelial cells in the vasculature. **(B)** Healthy endothelial glycocalyx showing proteoglycan backbone molecules attached to glycosaminoglycan chains.

The endothelial glycocalyx is complex and was difficult to visualize. J.H Luft was likely the first to visualize and describe the endothelial glycocalyx more than 5 decades ago with an electron microscope (Luft, 1966). In the recent years, the endothelial glycocalyx has gained a lot of interest especially as an important feature regulating vascular and blood physiology and an early marker of cardiovascular disease. The endothelial glycocalyx is about 0.5–4.5 μm thick increasing with vascular diameter from capillaries to the carotid arteries (Vink and Duling, 1996; van Haaren et al., 2003; Megens et al., 2007) and covers the surface of endothelial cells, virtually separating them from coming into contact with blood cells, an important physiological characteristic that ensures maintenance of hemodynamic homeostasis, Figure 1A.

The endothelial glycocalyx is rich in carbohydrate sugars with mainly proteoglycan and glycoprotein backbone molecules that connect and communicate with vascular endothelial cells (Foote et al., 2022). The endothelial glycocalyx is not fixed but active and soluble allowing for continuous interaction with plasma and blood flowing continuously in the vascular bed hence, constantly modulating its thickness. The endothelial glycocalyx is negatively charged, allowing a red blood cell exclusion zone or region of separation on the luminal side of the endothelium due to the repelling effect between the negatively charged red blood cell membrane and endothelial glycocalyx (Sembajwe et al., 2023). This protects the glycocalyx from shedding or damage. The proteoglycan component of the glycocalyx is the most important backbone molecule attached to glycosaminoglycan chains and is composed of syndecans and glypicans also known as glycosylphosphatidylinositol anchors (Carey, 1997; Fransson et al., 2004; Reitsma et al., 2007) and additional types of secreted soluble proteoglycans like perlecan, versican, mimecan, and biglycan (Iozzo, 1994; Kinsella et al., 2004; Reitsma et al., 2007), Figure 1B. The glycosaminoglycan chains bound to proteoglycans include dermatan and keratan sulfate, heparan and chondroitin sulfate, and hyaluronan. Crucial in playing a role during inflammatory responses are special glycoproteins such as selectins, integrins and immunoglobulin superfamily, within the glycocalyx whose expression is regulated by cytokines and complex signaling pathways (Harjunpää et al., 2019; Qu et al., 2021).

We have previously described the endothelial glycocalyx in detail (Sembajwe et al., 2023), the composition of the endothelial glycocalyx is well elaborated elsewhere (Reitsma et al., 2007; Villalba et al., 2021; Suzuki et al., 2022). We will therefore focus more on its physiological and pathological role in hypertension and HIV later below.

### 2.1 Physiological importance of the endothelial glycocalyx

The endothelial glycocalyx has several major functions in the vasculature. It regulates vascular permeability (Vink and Duling, 2000), restricts innate cells, red blood cells and other substances from coming into contact with the endothelium (Vink and Duling, 1996), but also facilitates the interaction of white blood cells to adhesion molecules during inflammatory responses, an important aspect of innate immunity. The endothelial glycocalyx also modulates antithrombotic and anticoagulant effects preventing coagulopathies and thrombotic events (Prior et al., 2017; Iba and Levy, 2019).

Apart from creating a physical barrier between the endothelium and contents of the vascular lumen, the endothelial glycocalyx also participates in mechanotransduction (Curry and Adamson, 2012). The endothelial glycocalyx also regulates blood viscosity and hematocrit, modulates shear stress and enhances flow-induced shear forces arising from shear-stress induced nitric oxide (NO) production (Mochizuki et al., 2003). The endothelial glycocalyx is also involved in the production of NO as removal of endothelial glycocalyx components was shown to inhibit the production of NO and impaired vasodilation (Pahakis et al., 2007; Dragovich et al., 2016). The mechanotransduction of the endothelial glycocalyx mediates NO production through Ca^2+^ entry and activation of endothelial transient receptor potential (TRP) channels (Dragovich et al., 2016). One mechanism for the endothelial glycocalyx mechanotransduction-induced production of NO is that during shear stress, the force applied to the components of the glycocalyx, syndecan and glypican-1, within the membrane caveolae, results in the phosphorylation of endothelial NO synthase (eNOS) by phosphoinositide-3-kinase and protein kinase A, leading to the activation of eNOS and production of NO (Boo et al., 2002). A study by Ebong et al demonstrated that silencing of glypican-1 abrogates eNOS activation and NO production suggesting that shear-induced production of NO was dependent on endothelial glycocalyx components (Ebong et al., 2010). Shear stress also regulates the activation of Ras-Mitogen-Activated Protein Kinases (MAPKs) which enhance cell survival mechanism by activating Krüppel-like factor 2 (KLF2) transcription factor and enhance the expression of syndecans as a protective measure to preserve the endothelial glycocalyx (Voyvodic et al., 2014). These data provide the premise for the shear stress sensing function of the endothelial glycocalyx.

As will be discussed later below, several factors that affect the integrity of the endothelial glycocalyx create a fertile ground for initial pathological processes that can escalate and increase the risk for cardiovascular disease and hypertension.

### 2.2 Determinants of endothelial glycocalyx integrity

The endothelial glycocalyx is susceptible to various factors that may underlie disease processes. These include but are not limited to chronic pro-atherogenic shear stress, interactive adverse influences from oxidized low density lipoprotein (Ahn et al., 2023), inflammatory cytokines such as tumor necrosis factor-α (TNF-α), interleukin-1 beta (IL-1β) and reactive oxygen species (ROS) that damage the glycocalyx by enhancing the activation of matrix metalloproteinases, heparanase, and hyaluronidase (Rubio-Gayosso et al., 2006; Uchimido et al., 2019). Traditional risk factors for cardiovascular disease such as hypertension, hyperglycemia, smoking, aging, bacterial and viral infections, and dietary salt contribute to the shedding of the endothelial glycocalyx leading to or exacerbating existing disease (Yamaoka-Tojo, 2020b; Patterson et al., 2022). Of all the determinants of the endothelial glycocalyx integrity, high salt intake and HIV infection are important contributors to endothelial glycocalyx shedding and consequently, to the development of hypertension and cardiovascular disease.

## 3 Sodium-endothelial glycocalyx interaction is the premise for endothelial dysfunction, hypertension, and cardiovascular disease

### 3.1 Sodium transport from the gut into the systemic circulation

After a salty meal, almost all the dietary salt is absorbed throughout the whole length of the small intestines in the jejunum and ileum under the control of angiotensin II and in the colon via the effects of aldosterone (Ryuzaki et al., 2022). Sodium uptake into the enterocytes across the brush border membrane (transcellular route) is made possible through several transporters such as Sodium/glucose cotransporter 1 (SGLT1), Na+ -dependent excitatory amino acid transporters, Na+ -dependent Ca2+ exchanger (NCX), Na+ -dependent hydrogen exchanger (NHE), Na+ -dependent potassium chloride cotransporter (NKCC), Na+ -dependent chloride cotransporter (NCC), Na+ -dependent magnesium exchanger (NME and Epithelial Sodium Channel (ENaC) (Gagnon and Delpire, 2021). Na+ is pumped out towards the blood stream by Na+/K + ATPase pump on the basolateral membrane (Wright and Loo, 2000).

Owing to the electronegative mucosal surface of the small intestines, Na+ also moves across from the lumen of the gut passively between the enterocytes (paracellular route) through the tight junctions into the interstitium and into portal circulation. In the vascular bed, the endothelial glycocalyx being negatively charged buffers Na+ ions but when in excess, this buffering capacity is reduced resulting in diminished red blood cell-endothelial glycocalyx repelling effect (Oberleithner et al., 2011; Kusche-Vihrog and Oberleithner, 2012). This leads to shedding of the glycocalyx allowing red blood cells to come into contact with endothelial cells, which activates them and stimulates endothelial cells to express adhesion molecules, favoring leukocyte adhesion (Langer and Chavakis, 2009; Jiang et al., 2021). Thus, a damaged glycocalyx promotes leukocyte adhesion and diapedesis as well as platelet recruitment that favors both inflammatory and thrombotic processes (Iba and Levy, 2019; Uchimido et al., 2019). Endothelial glycocalyx damage and endothelial cell activation increases vascular permeability allowing Na+ to extravasate and collect in the interstitial tissue where it induces inflammatory responses and edema (Nijst et al., 2015; Li et al., 2022; Sulyok et al., 2022). When the Na+ in the endothelial glycocalyx accumulates and increases in the luminal surface of the endothelial cells, this increases the activation and activity of the ENaC. ENaC activation leads to more sodium getting pumped into the endothelial cells and this causes stiffness of the cell’s cortex and reduces nitric oxide production especially with subsequent elevation of vascular tone (Kusche-Vihrog et al., 2014). These events contribute to the development of salt-sensitive hypertension (Sulyok et al., 2022), Figure 2.

**FIGURE 2:**
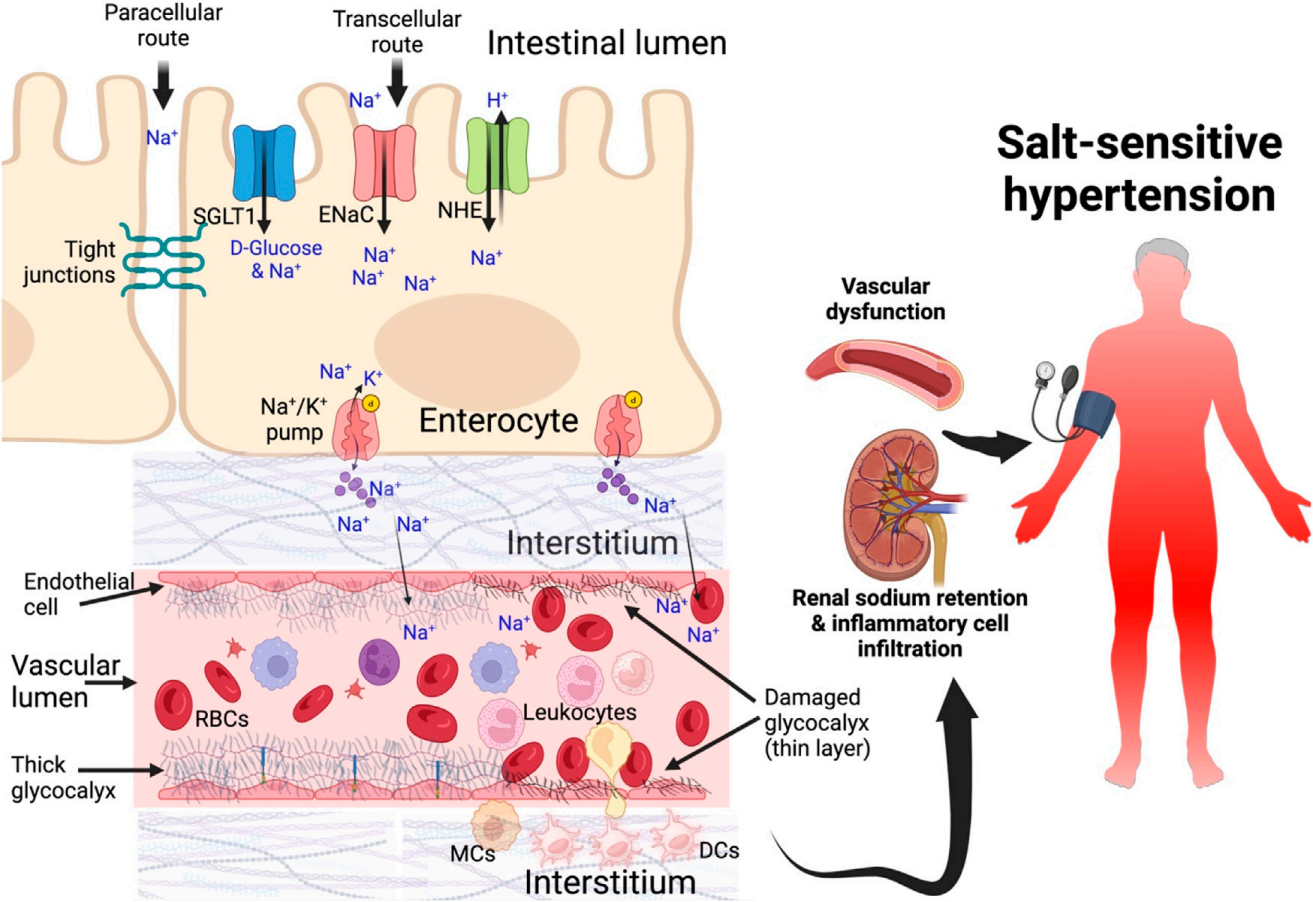
Sodium transport from the gut into blood circulation in salt-sensitive hypertension Sodium is transported from the intestinal lumen through enterocytes via sodium channels and across basolateral membrane into the interstitium and then into systemic circulation. As sodium increases in the interstitial spaces it is transported into the lumen. Moreover, dendritic cells in the interstitial spaces take up sodium and become activated, secreting inflammatory cytokines. Damaged glycocalyx allows sodium to extravasate and contribute to endothelial dysfunction, impair vascular tone, cellular infiltration of kidney and inflammation, sodium retention in the kidney leading to salt-sensitive hypertension. DCs, dendritic cells; SGLT1, sodium glucose transporters; ENaC, epithelial sodium channel; NHE, Na+ -dependent hydrogen exchanger.

### 3.2 High salt intake impairs endothelial glycocalyx leading to endothelial dysfunction, hypertension, and cardiovascular disease

Vinaiphat et al recently demonstrated that mice feed with high salt diet caused severe shedding and damage to the endothelial glycocalyx and induced hypertension suggesting that dietary salt directly contributes to the shedding of the endothelial glycocalyx, and disrupting the vascular integrity of multiple organs (Vinaiphat et al., 2023). High salt also impaired glycocalyx protein mediators involved in contractility, renal function, mechanotransduction, and coagulation cascades. Vinaiphat and colleagues further demonstrated that high salt promoted arterial wall inflammation due to infiltration by monocytes and macrophages. Monocyte infiltration in the arterial wall was mediated by increased expression of vascular adhesion protein-1 (VCAM-1) induced by high Na^+^ (Afsar et al., 2018).

In human studies, we have previously demonstrated that high salt intake correlates with poor erythrocyte glycocalyx sensitivity to sodium (eGCSS) (Masenga et al., 2022), a surrogate test for assessing endothelial glycocalyx integrity or damage based on a “salt blood test” (Oberleithner, 2015). In salt -sensitive hypertension, high Na+ reduces the production of NO and induces endothelial secretion of pro-inflammatory cytokines IL-1ß and TNFα (Vinaiphat et al., 2023). Damaged endothelial glycocalyx in the kidney correlates positively with endothelial dysfunction and uraemic toxins (Liew et al., 2021). Conversely, endothelial dysfunction is an independent predictor of atherosclerosis, hypertension and cardiovascular disease (Hadi et al., 2005; Brandes, 2014; Widmer and Lerman, 2014). In a study evaluating the relationship between endothelial glycocalyx damage and acute coronary syndrome, Miranda et al. (2016) found that serum levels of syndecan-1 were elevated in acute coronary syndrome. In another clinical study by Bielecka-Dabrowa et al. (2013), they found serum levels of syndecan-4 correlated negatively with left ventricular ejection fraction (LVEF) but positively with left ventricular systolic and diastolic diameters in patients with dilated cardiomyopathy. In several other studies, damaged endothelial glycocalyx was associated with acute or chronic heart failure and death (Tromp et al., 2014; Neves et al., 2015; Demissei et al., 2016).

Depending on the underlying cause, endothelial glycocalyx damage can either be systemic or localized. For example, in sepsis, bacteria toxins and inflammation can promote systemic glycocalyx thinning that may promote increased vascular permeability leading to systemic edema, hypovolemia and circulatory dysfunction resulting in multiorgan damage (Iba and Levy, 2019; Uchimido et al., 2019). However, there is scarcity of data that reports the extent of glycocalyx damage brought upon by HIV and dietary salt.

Although chronic high salt impairs endothelial glycocalyx, acute salt loading does not appear to affect or impair the endothelial glycocalyx (Vinaiphat et al., 2023). In a recent study we found that individuals with an immediate pressor response to oral salt (IPROS) had better endothelial glycocalyx scores, measured using a salt blood test (Masenga et al., 2022). However, the underlying reasons for this were unknown and beyond the scope of the study.

It takes approximately 5–7 days for a damaged endothelial glycocalyx to be restored *in vivo* (Potter et al., 2009). Rosemary and colleagues were able to show that plasma is able to restore the endothelial glycocalyx and preserve syndecan-1 after hemorrhagic shock (Kozar et al., 2011). Therapeutic approaches to restoring the endothelial glycocalyx are described in Section 6.

## 4 HIV infection, antiretroviral therapy and endothelial glycocalyx integrity, premise for endothelial function and cardiovascular disease

### 4.1 Endothelial glycocalyx integrity in persons with HIV

Currently there is dearth of literature linking endothelial glycocalyx damage and HIV infection. However, there are many studies that discuss endothelial function and the role of HIV in impairing endothelial function. In this section, we will attempt to bridge the gap by proposing mechanisms underlying damage to the endothelial glycocalyx that is mediated by HIV viral proteins and we will also discuss the role of ART on endothelial glycocalyx.

HIV has evolved to use glycocalyx components such as syndecans as attachments receptors allowing interaction (Saphire et al., 2001). The negatively charged endothelial glycocalyx enables binding of HIV viral protein portions that are positively charged and in addition hydrogen bonding and hydrophobic interactions between viral proteins and glycoepitopes ensures tighter binding (Cafaro et al., 2021; Foote et al., 2022; Fragkou et al., 2023).

HIV infection and high viremia is associated with damaged endothelial glycocalyx (determined by measuring perfused boundary region) which is only improved after initiating ART (Triantafyllidi et al., 2022; Fragkou et al., 2023). Compared to heathy individuals, PLWH have higher plasma Syndecan-1, a marker of damaged endothelial glycocalyx, and damage to endothelial glycocalyx was associated with markers of kidney dysfunction (Meneses et al., 2017). When PLWH were followed for a longer period (5 years) to determine the link between endothelial glycocalyx damage and kidney dysfunction, Cavalcante et al found that endothelial glycocalyx determined by plasma syndecan-1 levels worsened with chronic use of ART and correlated positively with worsening of creatinine and glomerular filtration rate (GFR) values (Gadelha Cavalcante et al., 2022). These findings have been reproduced in other studies (De Francesco Daher et al., 2016; Yamaoka-Tojo, 2020a; Fragkou et al., 2023) albeit very scarce in the HIV population.

### 4.2 Endothelial dysfunction in HIV

HIV-1 has limited specific cell types it can infect due to specific signature receptors such as CD4 and the co-receptor C-C chemokine receptor type 5 (CCR5) it can interact with to establish infection (Llorente García and Marsh, 2020; Weichseldorfer et al., 2022). Several cells/organs have been demonstrated to establish HIV-1 infection, we have evidence that HIV-1 can infect endothelial cells of the microvascular system, brain, liver and glomeruli via HIV viral particles without cytolysis (Lafon et al., 1993; Moses et al., 1993; Bagasra et al., 1996). *In vitro* experiments demonstrated that infected T-cells induce expression of intracellular adhesion molecule-1 and through IFN-γ enhance the adhesion of infected T-cells to endothelial cells activating them while viral replication in infected endothelial cells is enhanced by TNF-α and IL-1, events that all lead to endothelial dysfunction (Conaldi et al., 1995; Dianzani et al., 1996).

Viral particles are believed to play a critical role in shedding endothelial glycocalyx largely attributed to HIV viral proteins such as envelope glycoprotein (gp120), transactivator of transcription (Tat), and Nef which are secreted into- and interact with the endothelial glycocalyx during HIV infection (Anand et al., 2018; Spillings et al., 2022). Viral proteins mostly cause endothelial dysfunction mainly mediated by inflammatory responses. Nef and gp120 can enter into endothelial cells leading to endothelial dysfunction, oxidative stress, increased expression of adhesion molecules and inducing innate immune responses and inflammation (Arese et al., 2001; András et al., 2003; Avraham et al., 2004; Kanmogne et al., 2007).

Endothelial function including distensibility and arterial wall stiffness are mainly determined by flow mediated dilatation (FMD) which directly measures the dilatory effects of NO production by assessing change in brachial artery diameter before and after inducing reactive hyperemia. Determination of the endothelial glycocalyx is not necessarily a measure of endothelial function rather, an early indicator likely to cause endothelial dysfunction. However, serum levels of soluble syndecan-1 were independently associated with FMD suggesting its importance as a surrogate marker of endothelial function (Salmito et al., 2015). Many studies have demonstrated evidence of an impaired endothelial dysfunction measured by FMD among PLWH (Bonnet et al., 2004; Blum et al., 2005; Charakida et al., 2005; Solages et al., 2006). HIV viral load and ART use especially of the class of protease inhibitors, were independent predictors of endothelial function in PLWH (Bonnet et al., 2004; Blum et al., 2005; Charakida et al., 2005; Solages et al., 2006).

## 5 Mechanisms of endothelial glycocalyx damage, endothelial dysfunction and associated complications

### 5.1 Glycocalyx and endothelial cell mechanisms of damage

When the glycocalyx is shedding due to damage by infectious agents or other substances, fragments released into circulation serve as damage or danger-associated molecular patterns (DAMPs) and are recognized by pattern recognition receptors (PRR) on innate immune cells, eliciting suboptimal inflammatory response and trauma induced coagulopathy (TIC) within the vasculature (Johansson et al., 2011). High salt is a common cause of endothelial glycocalyx damage that impairs endothelial function. The endothelial glycocalyx participates in the activation of NO via mechanotransduction pathways to induce vasodilatory effects that serve to reduce blood pressure. Therefore, direct damage of the endothelial glycocalyx by salt-induced ROS causes endothelial dysfunction, diminishing vasodilatory function during salt-induced hypertension also seen in pre-eclampsia and increasing the risk for cardiovascular disease development (Bartosch et al., 2017; Schierke et al., 2017; Weissgerber et al., 2019; Stanhewicz et al., 2021). Early during pre-eclampsia, the endothelial glycocalyx has been demonstrated to be diminished, increasing the potential for endothelial activation and vascular injury (Weissgerber et al., 2019). Circulating concentrations of glycocalyx degradation products are higher in pre-eclampsia compared to normotensive women (Carlberg et al., 2022).

HIV viral proteins are able to modify the endothelial cells, disrupting the mechanotransduction pathways and interfering with endothelial cell function to maintain homeostasis (Anand et al., 2018). HIV viral proteins are immunogenic and elicit inflammatory responses which cause damage to the endothelial glycocalyx exposing the endothelial cells. Soluble HIV gp120 has been reported to induce apoptosis in endothelial cells of coronary vessels via the co-receptor CXCR4, induce endothelial permeability leading to endothelial dysfunction and induction of pro-inflammatory cytokines IL-6 and IL-8 in endothelial cells (Fiala et al., 2004; Yang et al., 2009). Gp120 induces apoptosis by activating caspase-3, upregulating Bax and signaling through p38 mitogen-activated protein (MAP) kinase (Ullrich et al., 2000; Khan et al., 2007). Gp120 also enhances endothelial expression of adhesion molecules recruiting inflammatory cells that further disrupt and damage the endothelial glycocalyx. As will be discussed in the next section, HIV viral protein gp120 is known to reduce NO levels leading to abnormal vascular tone regulation and activation of platelets, leading to thrombotic events (Chatterjee and Catravas, 2008; Jiang et al., 2010). The whole mark of HIV viral proteins leading to endothelial glycocalyx damage and endothelial dysfunction is by direct interaction with glycocalyx, endothelial cells and inducing inflammatory responses that further disrupt endothelial function accelerating atherosclerosis and leading to hypertension and cardiovascular disease (Anand et al., 2018).

In summary, the damage to endothelial glycocalyx by both HIV viral proteins and dietary salt as an initial step prior to endothelial dysfunction is mutually exclusive. The presence of HIV undermines vascular integrity so much that high dietary salt intake exacerbates the pathogenesis leading to endothelial dysfunction and cardiovascular disease. Both dietary salt and HIV promote inflammation that contributes to vascular dysfunction and hypertension (Ruggeri Barbaro et al., 2021; Mutengo et al., 2022; Pitzer et al., 2022). However, the mechanisms are obviously different. The whole mark of endothelial glycocalyx damage is that it induces a proinflammatory phenotype and increased leukocyte adhesion and this has been demonstrated in cultured endothelial cells (McDonald et al., 2016).

### 5.2 Activation of NADPH oxidase, NF-κB pathway and NLRP3 inflammasomes

Excess sodium in the damaged glycocalyx and interstitium increases entry of sodium into the endothelial cells and dendritic cells or monocytes/macrophages, respectively, via ENaC (Barbaro et al., 2017; Mutchler and Kleyman, 2019; Ertuglu and Kirabo, 2022; Nemeth et al., 2022; Pitzer et al., 2022). Na+ is exchanged for Ca2+ by the Na+/Ca2+ exchanger and the increased entry of Ca2+ into the cell activates protein kinase C which phosphorylates NADPH oxidase enzyme increasing its activity (Datla and Griendling, 2010). NADPH oxidase produces reactive oxygen species (ROS) causing oxidant stress and phospholipid peroxidation leading to formation of immunogenic isolevuglandin (IsoLG)-protein adducts (Barbaro et al., 2017). Dendritic cells process IsoLG-protein adducts into major histocompatibility molecules and presents them to naïve CD4+ T-cells, activating them. Activated CD4+ T-cells infiltrate the kidney and secret inflammatory cytokines such as IL-17A, TNF-α, and interferon-gamma (IFN-γ) that damage the vasculature and kidney leading to salt-sensitive hypertension (Dixon et al., 2017; Rucker et al., 2018; Xiao et al., 2018; Ertuglu et al., 2022; Pitzer et al., 2022), Figure 3. Although dendritic cells are referred to here, other cell types such as monocytes, macrophages and lymphocytes are also affected by high salt, contributing to immune activation and inflammation in salt-sensitive hypertension in both human and animal models (Zhang et al., 2015; Ren and Crowley, 2019; Sahinoz et al., 2021; Nemeth et al., 2022).

**FIGURE 3:**
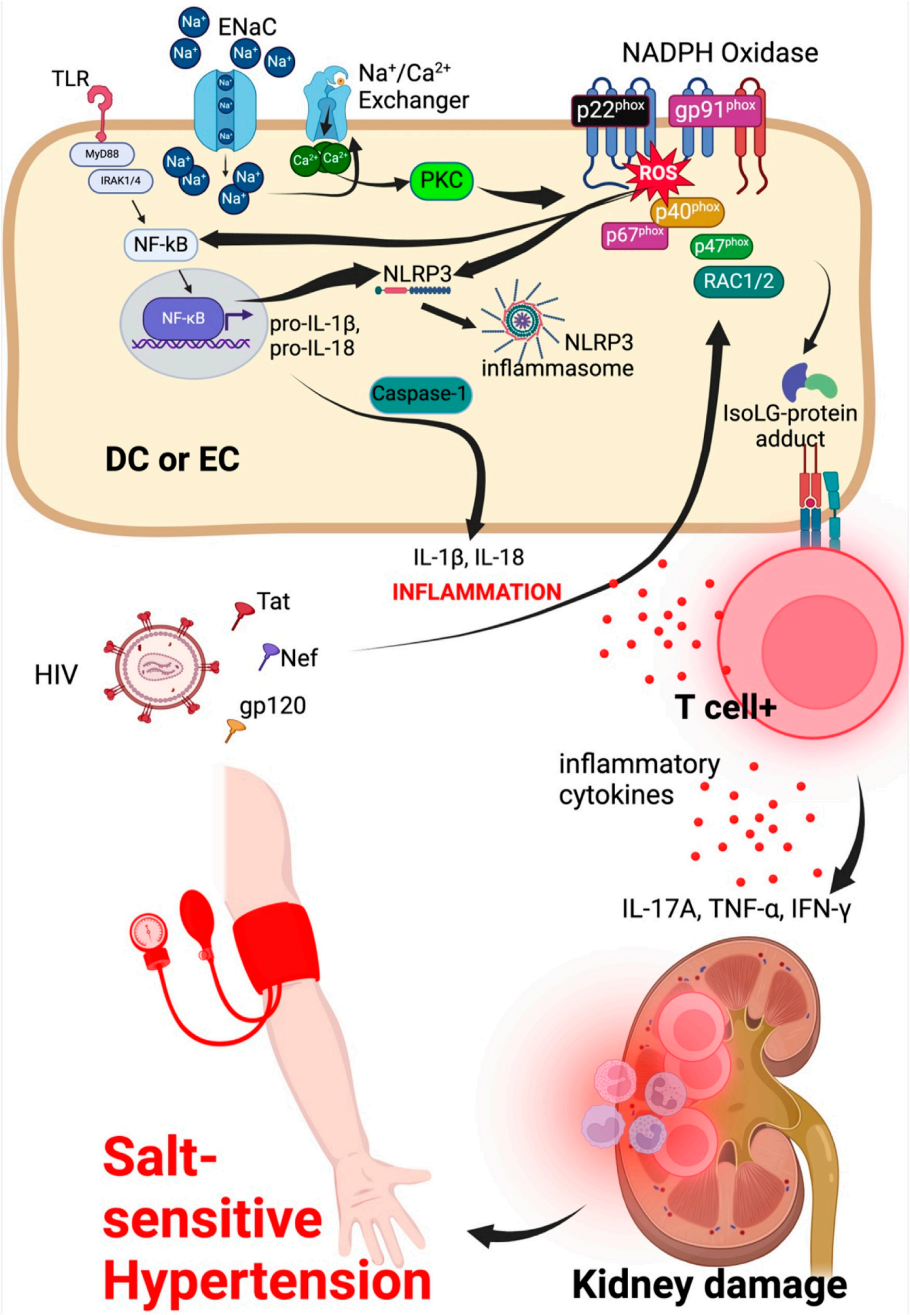
Sodium and HIV induced hypertension signaling mechanisms in dendritic or endothelial cell. Endothelial cells/dendritic cell combined for ease to explain entry of sodium and HIV viral particle effects on endothelial cell to activate intracellular mechanisms contributing to kidney damage and hypertension. ENaC, epithelial sodium channel; NLRP3, NLR family pyrin domain containing 3; NF-κB, Nuclear factor kappa-light-chain-enhancer of activated B-cells; MyD88, Myeloid differentiation primary response 88; TNFR, tumor necrosis factor receptor; TLR, toll-like receptor; IFN-γ, interferon gamma; DC, dendritic cell; EC, endothelial cell.

ROS also activate the nuclear factor kappa-light-chain-enhancer of activated B (NF-κB) which translocate into the nucleus to induce transcription of pro-inflammatory cytokines IL-1β and IL-18 (Pitzer et al., 2022). NF-κB also activates NLR Family Pyrin Domain Containing 3 (NLRP3) inflammasome which acts through caspase-1 component to cleave pro-inflammatory cytokines IL-1β and IL-18 into active IL-1β and IL-18. Activation of NF-κB and NLRP3 inflammasome is associated with production of tumor necrosis factor-alpha (TNF-α), IL-1β, and IL-6 inflammatory cytokines leading to vascular damage, endothelial dysfunction resulting in hypertension and cardiovascular disease (Ertuglu et al., 2022; Pitzer et al., 2022; Masenga et al., 2023). The NF-κB pathway is also activated by shear stress of the endothelial glycocalyx via activation of Rho family GTPases which in turn activates and induces the transcriptional activity of NF-κB (Li et al., 2005).

In HIV infection, damaged endothelial glycocalyx increases interaction between HIV viral proteins and endothelial cells. The viral proteins gp120, Tat and Nef impair ROS regulation. Tat induces activation of small GTPase Rac1/2 which phosphorylates a subunit of the dissociated NADPH oxidase, p47phox causing it to translocate to the membrane and merge with additional subunits p67phox, p40phox, gp91 phox and p22 phox, activating the NADPH oxidase and increasing its activity. NADPH oxidase is made up of two membrane subunits (p22phox and gp91phox) and four cytosolic subunits (p40phox, p47phox, p67phox and Rac1/2 (Rueckschloss et al., 2003; Belambri et al., 2018; Margaritis, 2019), Figure 3. Downstream signal transduction pathways that are activated owing to the activity of the NADPH oxidase include the Jun N-terminal kinase (JNK), extracellular signal-regulated kinase (ERK) and NF-kB pathways, promoting inflammatory responses by mechanisms already explained. Compared to dietary salt (sodium to be specific) which reduces NO generation via ENaC-protein kinase C-NADPH oxidase activation to produce ROS that directly inhibit NO generation, HIV viral protein gp120, Tat and Nef downregulate eNOS mRNA transcription and translation leading to reduced levels of the enzyme and consequently reducing the production of NO required to maintain vascular tone during hypertension (Margaritis, 2019).

### 5.3 Oxidative stress and enzymes directly damage the glycocalyx by fragmentation of its components

Oxidative stress is a key feature of endothelial dysfunction and results from an imbalance between enzymes that generate ROS (NADPH oxidase and eNOS) and antioxidant scavengers to counteract and destroy ROS in the vasculature (Münzel et al., 2017). Normally, when vascular tone increases, NO production is increased via conversion of L-arginine to L-citrulline by enzymatic activity of endothelial nitric oxide synthase, this leads to vasodilation of blood vessels, ameliorating high blood pressure (Loperena and Harrison, 2017; Ray et al., 2023). However, the high production of ROS resulting from increased NADPH oxidase activation (a result of increased Na+ entry into endothelial cells via ENaC) causes ROS to react directly and inhibit nitric oxide production. This impairs vasodilatory function, a characteristic feature of endothelial dysfunction in salt -sensitive hypertension (Datla and Griendling, 2010; Patik et al., 2021; Ruggeri Barbaro et al., 2021; Ertuglu et al., 2022).

ROS-induced production by inflammatory cells and endothelial cells directly damage and modify the endothelial glycocalyx increasing leukocyte adhesion and inflammatory responses (Rubio-Gayosso et al., 2006). Moreover, circulating fragments of damaged glycocalyx are immunogenic, thus, exacerbating the immune response and contribute to salt-sensitive hypertension (van Golen et al., 2012). The endothelial glycocalyx is very fragile to enzymatic (hyaluronidase, metalloproteinases, and heparinase) and ROS degradation. Metalloproteinases are secreted by endothelial cells, innate immune cells and smooth muscle cells when stimulated by inflammatory cytokines, shear stress, ROS and hypoxia (Hobeika et al., 2007; Castro et al., 2008; Iba and Levy, 2019). Metalloproteinases mainly degrade extracellular domains of syndecans and chondroitin sulfate (Dreyfuss et al., 2009; Sieve et al., 2018; Bogner-Flatz et al., 2019) while heparinase, which is secreted by endothelial cells, inflammatory cells and platelets cleaves the inner chains of heparan sulfate under the stimulation of ROS, hyperglycemia, advanced glycosylation products and inflammatory cytokines (Ishai-Michaeli et al., 1990; Chen et al., 2004; Han et al., 2005; Kramer et al., 2006; An et al., 2011; Masola et al., 2018).

### 5.4 Renal dysfunction and endothelial glycocalyx

In the kidney, ROS damage the glycocalyx contributing to kidney disease and hypertension (Singh et al., 2013). Oxidative stress in salt-sensitive hypertension is exaggerated as demonstrated by levels of urine F2-isoprostanes during low and high salt diets (Beltowski et al., 2004; Al-Solaiman et al., 2009). In the kidney, damaged endothelial glycocalyx induced by ROS and inflammatory cytokines increases production of endothelin-1 from activated endothelial cells contributing to vasoconstriction, inflammation and fibrosis (Rossi et al., 1999; Clozel and Salloukh, 2005; Neuhofer and Pittrow, 2006).

## 6 Diagnostic and therapeutic approaches to vascular glycocalyx

### 6.1 *In vitro* diagnostic visualization techniques

It is very difficult to develop direct visualization techniques to study the glycocalyx because its highly vulnerable to reagents. However, through the decades since 1966, several protocols have been developed to aid in visualizing the glycocalyx via transmission electron microscopy (TEM) using various reagents such as ruthenium red, Alcian blue 8GX, and fluorocarbon-based oxygen carrying fixatives (Luft, 1966; Rostgaard and Qvortrup, 1997; Rostgaard and Qvortrup, 2002; van den Berg et al., 2003). Apart from TEM, other visualization techniques that are more or have similar robustness can be employed such as confocal laser scanning microscopy (CLSM) (Barker et al., 2004) and two-photon laser scanning microscopy (Megens et al., 2007). TEM cannot be used *in vivo*.

Several direct approaches have been employed using proteins that bind specific glycocalyx components. Examples include use of lectins to bind components of glycosaminoglycan chains (Florian et al., 2003; Barker et al., 2004; Mulivor and Lipowsky, 2004) and use of antibodies that bind to specific glycocalyx components such as syndecan, hyaluronan and heparan sulfate (Florian et al., 2003; Mulivor and Lipowsky, 2004). The use of antibodies are particularly important to target specific components of the glycocalyx. Puerta-Guardo and colleagues used antibodies in Immunofluorescence staining to study endothelial glycocalyx using human pulmonary, dermal, and umbilical vein microvascular endothelial cells (Puerta-Guardo et al., 2016). The antibodies used in studying the glycocalyx included anti-human cathepsin L, anti-human heparanase 1, anti-human CD138 for syndecan-1, anti-heparan sulfate proteoglycan two for perlecan among others. For imaging modalities, they used inverted fluorescence microscopy. Several other studies have used antibodies to visualize the endothelial glycocalyx (McDonald et al., 2016; Puerta-Guardo et al., 2016; Butler et al., 2019; Wang et al., 2019).

Several *in vitro* techniques have been described elsewhere (Haymet et al., 2021) and will therefore not be repeated here.

### 6.2 *In vivo* experimental visualization techniques

In 1996, Vink and colleagues were able to indirectly visualize the endothelial glycocalyx using intravital microscopy in muscle capillaries of hamster cremaster (Vink and Duling, 1996). They labeled plasma with a fluorescent dextran so that the plasma exclusion zone represented the glycocalyx area. This method has been used by many researchers onwards using both hamsters (Henry and Duling, 1999; 2000; Vink and Duling, 2000) and mice (Constantinescu et al., 2003; Rubio-Gayosso et al., 2006) but is an indirect technique of visualizing the glycocalyx.

Two-photon laser scanning microscopy can be used for both *ex vivo* and *in vivo* visualization of the glycocalyx with good resolution (Megens et al., 2007).

More recently, the sublingual microcirculation with its glycocalyx has been visualized using the glycoCheck system using a camera and dedicated software with moderate reproducibility (Eickhoff et al., 2020; Bol et al., 2022). The glycoCheck system measures the perfused boundary region to determine glycocalyx thickness, and is also able to determine red blood cell filling percentage, and microvascular vessel density (Eickhoff et al., 2020; Bol et al., 2022).

Surrogate tests for assessing endothelial glycocalyx integrity are available. Determination of plasma levels of Syndecan-1 can be used as a marker of endothelial glycocalyx. Syndecan-1 could also be used as a marker of renal dysfunction in PLWH where it correlates positively with serum levels of creatinine and urea (Meneses et al., 2017). Syndecans are more important and clinically relevant to determine as they are the main components of the endothelila glycocalyx correlating with human health and disease.

### 6.3 Current therapeutic approaches

Several experimental and non-experimental approaches to restore endothelial glycocalyx have been described. Use of spironolactone has been demonstrated to reduce endothelial glycocalyx deterioration and restore endothelial function via mineralocorticoid receptor antagonism (Schierke et al., 2017). In animal models, use of hydrocortisone not only protected rats against ischaemia-reperfusion injury but prevented damage and degradation of the endothelial glycocalyx (Chappell et al., 2007; Gao et al., 2015). Human studies to modulate endothelial glycocalyx remain scarce. However, several therapies are being repurposed to induce endothelial glycocalyx regeneration. Examples of drug therapies include artersil, endocalyx, rosuvastatin, sulodexide, hydrocortisone, metformin, etanercept, antithrombin, poloxamer, albumin, fresh-frozen plasma, hydroxyethyl starch, and heparin, these have discussed in detail by Banerjee et al. (2021) and Masola et al. (2021).

In PLWH, use of an integrase strand transfer inhibitor such as dolutegravir, raltegravir or elvitegravir/cobicistat is associated with restoration of endothelial glycocalyx integrity (Fragkou et al., 2023).

## 7 Future perspectives

More studies are required to bridge the gap and link between high salt and HIV in promoting endothelial glycocalyx degradation, endothelial dysfunction and hypertension. There are currently no studies known to us that have investigated the synergistic role contributed by dietary salt and HIV in the study of the endothelial glycocalyx.

## 8 Conclusion

High salt intake damages the endothelial glycocalyx inducing salt sensitive hypertension. Moreover, it is also well established that PLWH have a damaged endothelial glycocalyx due to HIV viral proteins and use of ART which is associated with subsequent endothelial dysfunction and development of cardiovascular disease. Although salt sensitive hypertension is more prevalent in PLWH, the role of dietary salt and HIV-related factors in contributing to endothelial glycocalyx damage are limited.
